# A Rare Case of Bilateral Abdominoscrotal Hydrocoele in a 10-Month-Old Infant Managed Laparoscopically

**DOI:** 10.7759/cureus.24875

**Published:** 2022-05-10

**Authors:** Zakaria W Shkoukani, Sarah N Aldhefeeri, Raed Al-Taher

**Affiliations:** 1 General Surgery Department, Aberdeen Royal Infirmary, Aberdeen, GBR; 2 General Surgery Department, Jordan University Hospital, Amman, JOR

**Keywords:** general paediatric surgery, minimal access surgery, laparoscopic treatment, bilateral hydrocoele, abdomino-scrotal hydrocoele

## Abstract

Abdominoscrotal hydrocoeles (ASH) are an increasingly rare form of hydrocoeles. They can present in any age group; however, they are more commonly reported in the paediatric population. Although not yet confirmed, the aetiology of ASH has been under scrutiny for the past two centuries, and scarcity of reported cases hinders this process. Clinical examination is oftentimes sufficient to make the diagnosis; however, confirmatory ultrasonography is recommended. Although old reports favoured a more conservative approach with watchful waiting, the risk of serious secondary complications is high, and surgical intervention is hence considered standard of care. Different approaches have been described, each with their own benefits, with minimally invasive surgery becoming more prevalent as of late. A case of a 10-month-old boy with bilateral ASH treated with a laparoscopic technique is presented.

## Introduction

A hydrocoele is a cystic mass filled with fluid that develops between the two layers of the tunica vaginalis, the serous membrane that covers the testes [1]. Hydrocoeles can arise due to a variety of reasons in both the paediatric as well as the adult population, rendering a specific classification essential to differentiating the cause in each case and hence choosing appropriate management. Hydrocoeles can be classified into primary, secondary communicating, secondary non-communicating, congenital, and tumour-induced, with many other types and sub-types [1,2].

Notably, abdominoscrotal hydrocoeles (ASH) are renowned for being the rarest of all hydrocoele forms, and they typically involve a dumbbell-shaped collection which extends from the scrotum into the abdomen via the inguinal canal [1]. Contrary to other types, ASH cases are highly unlikely to resolve spontaneously, with secondary complications being potentially life-threatening. Surgical intervention is thus considered the current recommended standard for treatment [1-3].

We herein present a case of a 10-month-old boy with a bilateral abdominoscrotal hydrocoele who underwent laparoscopic surgical repair.

## Case presentation

A 10-month-old boy presented to the paediatric surgery outpatient clinic as a referral from his paediatrician. Prior to this presentation, the patient had been seen by his paediatrician and was found to have bilateral testicular swelling since birth. Upon being examined by the paediatrician, the swelling was found to be approximately 5x4cm in size and was translucent in nature. The infant was thus diagnosed with a bilateral hydrocoele, and expectant management was advised, with watchful waiting until one year of age. He was otherwise fit and well, with no significant past medical or surgical history.

Over the course of the next few months since paediatric assessment, the infant’s testicular swelling dramatically increased in size, warranting a review. Despite the patient being haemodynamically stable with no alarming local, gastrointestinal or urinary symptoms, the drastic increase in size of the swelling prompted a referral to paediatric surgeons for further assessment and investigation.

**Figure 1:**
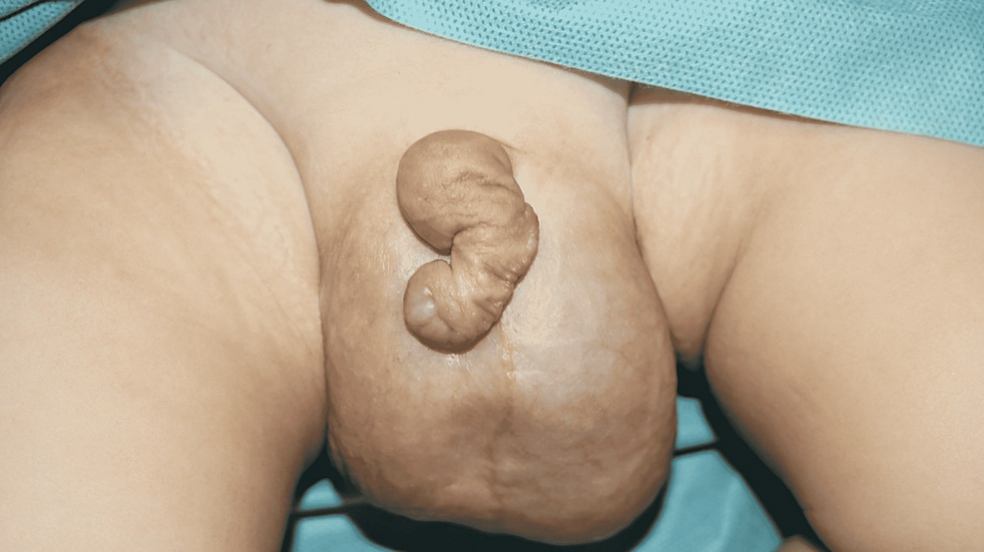
Pre-operative image of bilateral scrotal swelling and lymphoedema of the phallic skin and foreskin.

On examination in the paediatric surgery clinic, the 10-month-old boy was found to have remarkable swelling of the scrotum, with the left side being positive for translucency and the right side being negative for the same test. Neither of the testicles was palpable at this point in time. General examination was otherwise unremarkable. Marked lymphoedema of the phallic skin and foreskin was also noted, as can be seen in Figure 1.

**Figure 2:**
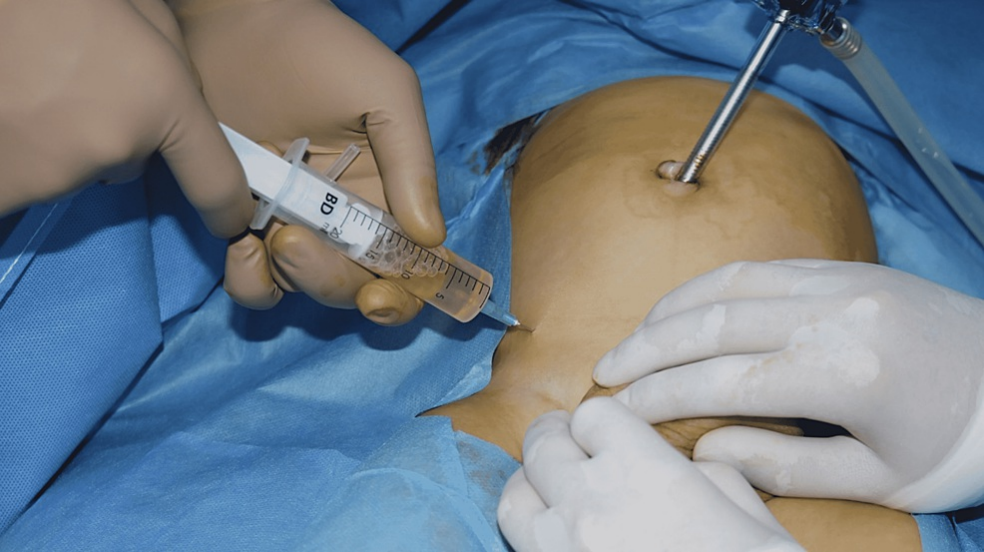
Percutaneous aspiration of right-sided hydrocoele.

The aforementioned findings warranted further investigation with scrotal ultrasonography, which revealed massive bilateral ASH (maximum length on left: 8.3cm, maximum length on right: 6.4cm) with a characteristic dumbbell appearance, homogenous anechoic content, and positive Doppler blood flow detected in both testes with no evidence of bowel herniation in either of the inguinal canals. The patient was thus scheduled for a laparoscopic procedure and admitted to the day-case unit. Laparoscopic procedure was performed under general anaesthesia with right-sided ligation of patent processus vaginalis (PPV) and hydrocoele percutaneous aspiration. Left-sided abdomino-inguinal hydrocoele was repaired in a similar fashion with aspiration and deroofing, as well as PPV ligation. Right-sided hydrocoele aspiration was quantified at 15 millilitres. Aspiration of the left-sided hydrocoele amounted to 100 millilitres. Bilateral PPV closure was performed using the LAPIRS (laparoscopic assisted percutaneous internal ring suturing) technique. Figure 2 shows the intra-operative percutaneous hydrocoele aspiration technique.

Whilst in the operating theatre, the patient also underwent circumcision upon patient parental request (as is customary in Jordan). It is worth noting that circumcision procedure was done irrespective of diagnosis and was not part of active management of the patient’s presenting condition. The procedure was performed successfully with no intraoperative complications, and appropriate haemostasis was ensured throughout. Post-operative recovery was satisfactory, with the patient being admitted overnight for prophylactic antibiotics and fluids. The patient was deemed fit for discharge the following day and has since improved remarkably with no signs of recurrence on routine follow-up.

## Discussion

A hydrocoele, in all its many shapes and forms, although frequently benign, can in many instances be the manifestation of a more lethal underlying condition. ASH in particular, being the least common variant, is the type known to most often present with significant complications if not rectified in due course [1].

A systematic review performed in 2018 by Gadelkareem included 320 studies, of which 299 were single case reports and 21 were case series. The total number of ASH cases was thus reported to be 579 cases, with 360 cases reported in paediatric patients and the remainder reported in adults. In paediatric cases, rates of ASH were found to range from 0.18% to 3.1% of all types of hydrocoeles. This systematic review described all cases between 1777 and2017, highlighting the rarity of the condition [4].

Although first identified in 1777 by Parcival-Pott, it was not until 1834 that Dupuytren postulated an aetiological explanation for ASH [4]. In his opinion, ASH originates from a pre-existing testicular hydrocoele, which accumulates over time leading to a build-up of pressure, eventually displacing the fluid through the inguinal canal and into the abdominal cavity [2,3]. However, with time, more cases were reported, allowing for a better understanding of the physical principles involved in the development of the condition, and thus new aetiological theories soon emerged. One theory emphasised the possibility of a one-way valve-like mechanism existing in a PPV at the deep inguinal ring. Two other theories include the diverticulum theory, wherein a peritoneal diverticulum herniates into the inguinoscrotal space, and the fluid imbalance theory, in which there is an imbalance in production and resorption of fluids. Despite prevalent controversy over the nature of ASH, the majority of authors in the literature, including ourselves, support Dupuytren’s hypothesis [3].

Identifying ASH as a diagnosis can, in many cases, be a challenge. Generally speaking, it should be suspected when a scrotal swelling is found in conjunction with an abdominal swelling that is localised around the area of the deep inguinal ring [2]. In the majority of cases, it presents unilaterally, with bilateral cases (as was the case in this report) presenting much less frequently [4]. The characteristic ‘springing back ball’ manoeuvre frequently described in the literature can be of use as a diagnostic aid. This involves scrotal compression, leading to a transient shifting of fluid from the scrotum into the abdomen, after which the swelling spontaneously springs back into the scrotum once more. Transillumination, commonly used in the diagnosis of hydrocoeles, can also be utilised to demonstrate an hour-glass swelling in a darkened room [5].

Ultrasonography is an easily accessible and commonly used method for further evaluation and is generally recommended for verification of the diagnosis [6]. Dynamic examination with compression of the scrotal component will demonstrate a patent communicating tract through the inguinal canal with an absence of bowel loops. This will effectively rule out an indirect inguinal hernia which is commonly mistakenly diagnosed in ASH cases [2]. Evaluation of testicular blood flow via duplex Doppler is also of significant importance in estimating the severity of the case at hand and deciding urgency of required intervention. Further investigation using CT and MRI may be warranted in a select few cases, especially in those who present with alarming urological or gastrointestinal manifestations [5,7,8].

Important differential diagnoses to rule out include spermatic cord lymphangioma, testicular tumour, severe cases of hydronephrosis that extend into the inguinal area, bladder diverticulae and pelvic neuroblastoma [9].

In comparison to other types of hydrocoeles, once a diagnosis of ASH is confirmed, intervention must be scheduled. This is due to the increased chances of secondary complications if left untreated. Complications of ASH include impacted spermatogenesis, sexual dysfunction and infertility, rupture and, consequently, haematocoele formation, hydronephrosis, appendicitis, lymphoedema, cryptorchidism, and malignant transformation to name a few [1,2,10].

Although different perspectives on the most suitable means of managing ASH exist, surgical intervention remains the standard of care for this condition [4]. Whilst some authors favoured a conservative approach to managing ASH, secondary complications are likely to arise in such situations and surgery is sometimes required, often as an emergency. Current methods of treatment as per the literature include traditional open surgery and minimally invasive total excision, with some authors still supporting alternative methods such as puncture and aspiration with or without sclerotherapy, or even watchful waiting. Others even resorted to a combination of two or three of these. Different approaches for operative intervention exist - inguinal, peritoneal, or scrotal approaches - depending on the size of the ASH [1,4].

Overall, the surgical procedure is considered to be a difficult one due to the fact that in many cases the hydrocoele attaches itself to the cord structures along with a thickened tunica vaginalis, which can be difficult to access regardless of technique. Aspiration of the fluid prior to dissection of the membrane can thus help facilitate easier access [2]. The use of laparoscopy in the treatment of ASH is becoming commonplace, with the added benefit of a confirmatory as well as a therapeutic role [11]. Laparoscopic approach was employed in our case and showed satisfactory results with no signs of recurrence on further routine follow-up.

## Conclusions

ASH is a rare form of hydrocoeles. Knowing the embryological origin as well as the common presenting features is pertinent to making an accurate diagnosis. Despite prevalent controversy over the true aetiology of this condition, simple manoeuvres employed during physical examination, alongside a confirmatory ultrasonography, can in most cases be sufficient to make a diagnosis. In doing so, appropriate surgical management can ensue and serious complications are thus avoided.

## References

[1] Dagur G, Gandhi J, Suh Y (2017). Classifying hydroceles of the pelvis and groin: An overview of etiology, secondary complications, evaluation, and management. Curr Urol.

[2] Costantino E, Ganesan GS, Plaire JC (2017). Abdominoscrotal hydrocele in an infant boy. BMJ Case Rep.

[3] Cuervo JL, Ibarra H, Molina M (2009). Abdominoscrotal hydrocele: its particular characteristics. J Pediatr Surg.

[4] R.A. Gadelkareem (2018). Abdominoscrotal hydrocele: a systematic review and proposed clinical grading. African Journal of Urology.

[5] Kamble PM, Deshpande AA, Thapar VB, Das K (2015). Large abdominoscrotal hydrocele: Uncommon surgical entity. Int J Surg Case Rep.

[6] Khalili M, Gholamzadeh Baeis M, Rouzrokh M (2020). Abdominoscrotal hydrocele: a case report. Urol Case Rep.

[7] Singh D, Aga P, Goel A (2011). Giant unilateral hydrocele "en-bisac" with right hydronephrosis in an adult: a rare entity. Indian J Urol.

[8] Yarram SG, Dipietro MA, Graziano K, Mychaliska GB, Strouse PJ (2005). Bilateral giant abdominoscrotal hydroceles complicated by appendicitis. Pediatr Radiol.

[9] Latabi A, Lakmichi MA, Dahami Z, Moudouni MS, Sarf I (2018). Giant abdomino scrotal hydrocele: a case report with literature review. Pan Afr Med J.

[10] Amodeo A, Liguori G, Trombetta C, Calgaro A, Patel HR, Belgrano E (2009). A giant heterogeneous abdominoscrotal mass: haemorrhagic hydrocele. J Radiol Case Rep.

[11] Funatsu Y, Shono K, Hashimoto Y, Shirai T, Shono T (2020). Laparoscopic abdominoscrortal hydrocele: a case series. Urology.

